# Low-Dose Phosphodiesterase III Inhibitor Reduces the Vascular Amyloid Burden in Amyloid-β Protein Precursor Transgenic Mice

**DOI:** 10.3390/ijms21072295

**Published:** 2020-03-26

**Authors:** Yusuke Yakushiji, Kazuhiro Kawamoto, Kazuyoshi Uchihashi, Masafumi Ihara, Shigehisa Aoki, Yukiko Nagaishi, Kohei Suzuyama, Yumiko Tsugitomi, Hideo Hara

**Affiliations:** 1 Division of Neurology, Department of Internal Medicine, Saga University Faculty of Medicine, Saga 849-8501, Japan; kawamotokazu@icloud.com (K.K.); suzusuzu61@gmail.com (Y.N.); sj8817@cc.saga-u.ac.jp (K.S.); hihara@cc.saga-u.ac.jp (H.H.); 2 Department of Pathology and Microbiology, Saga University Faculty of Medicine, Saga 849-8501, Japan; uchihashi.kazuyoshi.du@mail.hosp.go.jp (K.U.); aokis@cc.saga-u.ac.jp (S.A.); 3 Department of Neurology, National Cerebral and Cardiovascular Center, Suita 564-8565, Japan; ihara@ncvc.go.jp; 4 Department of Internal Medicine, Saga University Faculty of Medicine, Saga 849-8501, Japan; y.tsugitomi@gmail.com

**Keywords:** phosphodiesterase III inhibitor, cerebral micro-hemorrhage(s), cerebral amyloid angiopathy, amyloid-β protein, transgenic mice

## Abstract

A previous study reported that relatively high-dose cilostazol (0.3%) promoted the drainage of cerebrovascular amyloid-β (Aβ) protein in Aβ Precursor Protein (APP) transgenic mice overexpressing vasculotropic Aβ. We investigated whether lower-dose cilostazol can decrease micro-hemorrhages and Aβ deposition in the brain using APP transgenic mice. At baseline, 14-month-old female Tg2576 mice were randomly assigned to a control group (vehicle), aspirin group (0.01% aspirin), or cilostazol group (0.01% cilostazol). The severity of cerebral micro-hemorrhages (i.e., number), area of senile plaque, and severity of vascular amyloid burden (quantified with cerebral amyloid angiopathy (CAA) score (=number of Aβ-positive vessels × severity of amyloid burden of Aβ-positive vessels) were evaluated in the brain of mice aged 15 and 21–23 months. At 15 months, no differences were shown in each pathological change among the three groups. At 21–23 months, there were no differences in the severity of cerebral micro-hemorrhages or area of senile plaque among the three groups. However, the CAA score was significantly lower in the cilostazol compared to the control group (*p* = 0.046, Mann–Whitney *U* test), although no difference was seen between the control and aspirin group. Our study showed that lower-dose cilostazol could reduce the vascular amyloid burden without increasing cerebral micro-hemorrhages in APP transgenic mice.

## 1. Introduction

Sporadic cerebral amyloid angiopathy (CAA) is characterized by the progressive deposition of amyloid-β (Aβ) protein in the walls of small- to medium-sized arteries, arterioles, and capillaries in the cerebral cortex and overlying leptomeninges [1,2]. CAA is a common age-related cerebral small vessel disease (SVD) in the elderly [2,3] (especially those with Alzheimer’s disease [4]), but it is most often recognized clinically by symptomatic intracerebral hemorrhage (ICH) restricted to the lobar areas of the brain [5,6]. Long-term antiplatelet therapy, which is widely used for the secondary prevention of cerebral infarction, myocardial infarction, and peripheral artery diseases, could increase the incidence of ICH. In consideration of the high incidence of cerebral bleeding in patients with CAA and/or Alzheimer’s disease, especially in antiplatelet drug users [7,8], the safety of antiplatelet therapies for those patients should be explored.

Cyclic nucleotide phosphodiesterases (PDEs) play critical roles in regulating intracellular cyclic nucleotides (cyclic adenosine monophosphate (cAMP) and cyclic guanosine monophosphate), which are important secondary messengers involved in intracellular signal transduction in all tissues. PDE III is the major cAMP-hydrolyzing PDE (a negative regulator of cAMP) uniquely expressed in vascular smooth muscle cells. A selective inhibitor of PDE III, cilostazol, has multiple effects on the vasculature including vasodilatation, antioxidation, anti-inflammation, the regulation of smooth muscle cell, and an increase in cerebral hemodynamics, pulse duration time, and arterial elasticity with the maintenance of microvascular integrity [9]. Cilostazol is known as a unique antiplatelet drug, which is superior to aspirin in terms of safety for reducing ICH after an ischemic stroke [10]. Cilostazol ameliorates collagenase-induced cerebral hemorrhage by protecting the blood–brain barrier in mice [11]. In a CAA mice model (Tg-SwDI mice), cilostazol improved cognitive performance, which may be associated with reduced Aβ deposition by cilostazol (0.3% cilostazol) [12]. However, it is unknown whether the safety and efficacy of cilostazol could be replicated in different settings (i.e., drug dose or experimental transgenic mice). Thus, we sought to determine whether lower-dose cilostazol could reduce the incidence of cerebral (micro-)hemorrhages or cerebrovascular Aβ depositions using different transgenic mice as a CAA model.

## 2. Results

### 2.1. Survival Rate, Feed Consumption, and Drug Intake

**Figure 1:**
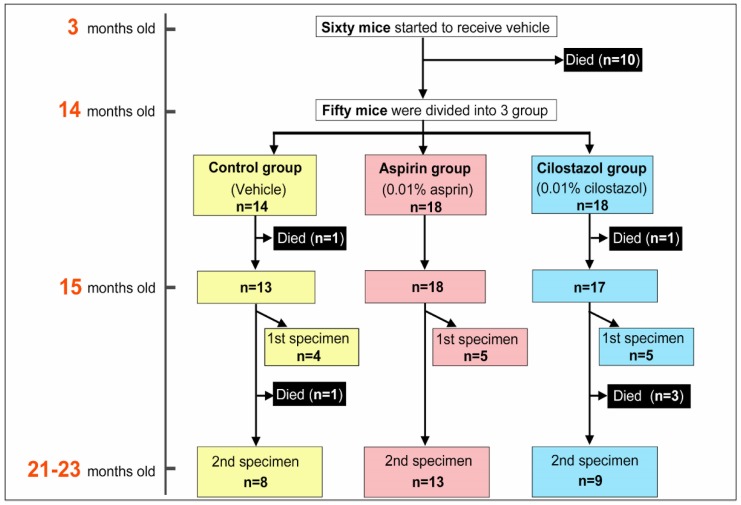
The flow diagram of study schedule and grouping.

**Figure A1:**
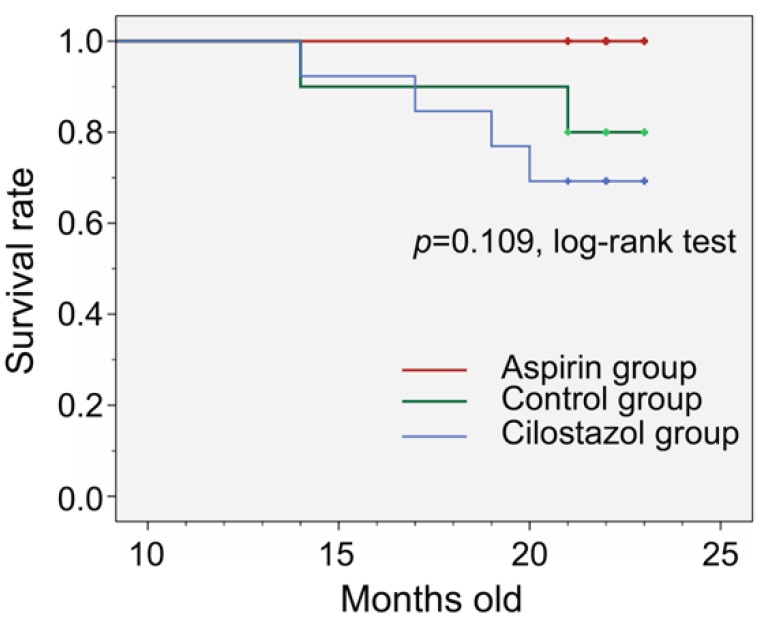
The survival rates after the grouping. There is no significant difference in the survival rate after the grouping among the three groups (control group, 80%; aspirin group, 100%; cilostazol group, 69% (*p* = 0.109, log-rank test: 14 mice evaluated for the first specimen are excluded from the analyses). Even in the two group comparisons, no differences are seen in the survival rate between the control group and the aspirin or cilostazol group (*p* = 0.098 and *p* = 0.550, log-rank test).

Sixty Tg2576 mice aged 3 months initially received the vehicle, but 10 mice died due to unknown reasons before 14 months. Thus, 50 mice aged 14 months were divided into three groups: control group (*n* = 14), aspirin group (*n* = 18), and cilostazol group (*n* = 18) (Figure 1). Within a month after the grouping, two mice died due to unknown reasons (control group, *n* = 1; cilostazol group, *n* = 1). Of the 48 survivors aged 15 months, 14 mice (control group, *n* = 4; aspirin group, *n* = 5; cilostazol group, *n* = 5) were randomly selected for the first evaluation (i.e., the first specimen). Among the remaining mice (*n*= 34), four mice died due to unknown reasons (control group, *n* = 1; cilostazol group, *n* = 3). Finally, 30 surviving Tg2576 mice aged 21–23 months were evaluated as the second specimens. There was no significant difference in the survival rate after the grouping among the three groups when the 14 mice evaluated for the first specimen were excluded from the analyses (*p* = 0.109, log-rank test: Figure A1: please see appendix). Even in the two group comparisons, no differences were seen in the survival rate between the control group and the aspirin or cilostazol group (*p* = 0.098 and *p* = 0.550, log-rank test). Table 1 shows estimated individual food consumption and drug intake (per mouse) of the three groups. There was no significant difference in food consumption between the control group (mean, standard deviation [SD]: 3.57 ± 0.40 g/day) and the aspirin group (3.67 ± 0.47 g/day) or the cilostazol group (3.67 ± 0.44 g/day) (*p* = 0.289 and *p* = 0.543, Mann–Whitney *U* test). Individual daily drug intake in the aspirin group (14.7 ± 1.9 mg/kg/day) was similar to that in the cilostazol group (14.7 ± 1.8 mg/kg/day).

### 2.2. Confirmation of Age-Related Cerebrovascular Amyloid Burden and Smooth Muscle Cell Loss

**Figure 2:**
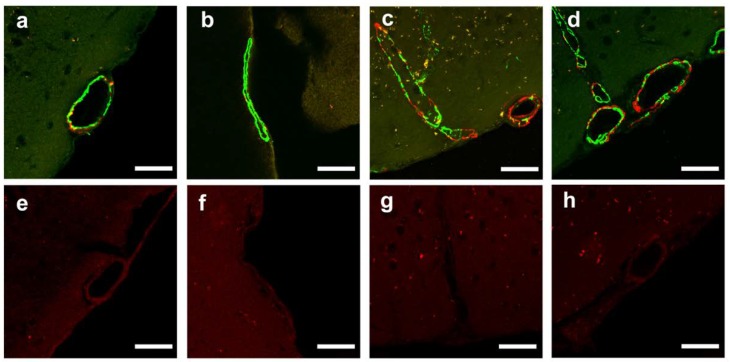
Upper line (**a**–**d**): Serial confocal microscopic changes of double-immunolabeled vessels (green, smooth muscle actin; red, amyloid). Lower line (**e**–**h**): Confocal microscopic findings represent negative controls without primary antibody for Aβ. a, b, Leptomeningeal vessel in a 15-month-old mouse shows small amyloid deposition and focal loss of smooth muscle cells at the site of cerebrovascular amyloid; c, d, In a 23-month-old mouse, smooth muscle cells are lost, and a thick sheet of amyloid covers the wall of a leptomeningeal vessel. e–f, Photos of the negative controls for Aβ (each section adjacent to a, b, c, d, respectively) show only a faint background stain in the vessel walls. Scale bar: 50 µm.

Confocal microscopic observation of double-immunolabeled vessels in different Tg2576 mice (15 months old, and 23 months old) fed with standard pelleted chow (i.e., control group) confirmed the age-related progression of amyloid burden and loss of smooth muscle cells (Figure 2a–d). The findings of the negative controls for Aβ show only a faint background stain in the vessel walls (Figure 2e–h).

### 2.3. Acute Subdural or Cerebral Bleeding(S)

**Figure A2:**
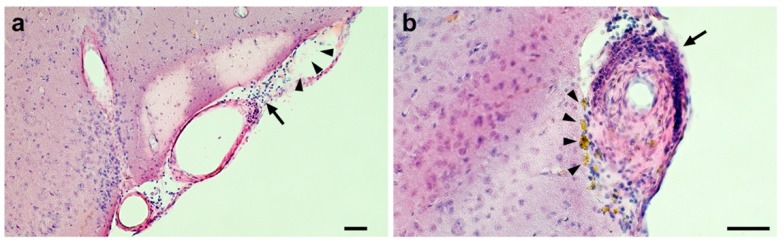
A representative image of accumulation of erythrocytes or hemosiderin. Accumulation of erythrocytes or hemosiderin shown in a Tg2576 mouse (aged 21 months old) fed with aspirin (i.e., aspirin group) (H&E stain). (**a**) Small accumulations of erythrocytes (arrow heads) are found around a leptomeningeal artery with lymphocytic infiltration (arrow). (**b**) Hemosiderin depositions (arrow heads) are seen around a leptomeningeal artery with vasculitis (arrow). Scale bars indicate 50 µm.

In both the first specimens (evaluated in mice aged 15 months) and the second specimens (evaluated in mice aged 21–23 months), no acute subdural or cerebral bleeding was found. However, in the second specimens in the aspirin group, small accumulations of erythrocytes were found in one mouse around a leptomeningeal artery with lymphocytic infiltration (Figure A2a). Hemosiderin depositions (arrow heads), representing old bleeding, were also seen around a leptomeningeal artery with vasculitis in the same mouse (Figure A2b). No accumulations of erythrocyte were found in either the control and the cilostazol group.

### 2.4. Cerebral Micro-Hemorrhages

**Figure 3:**
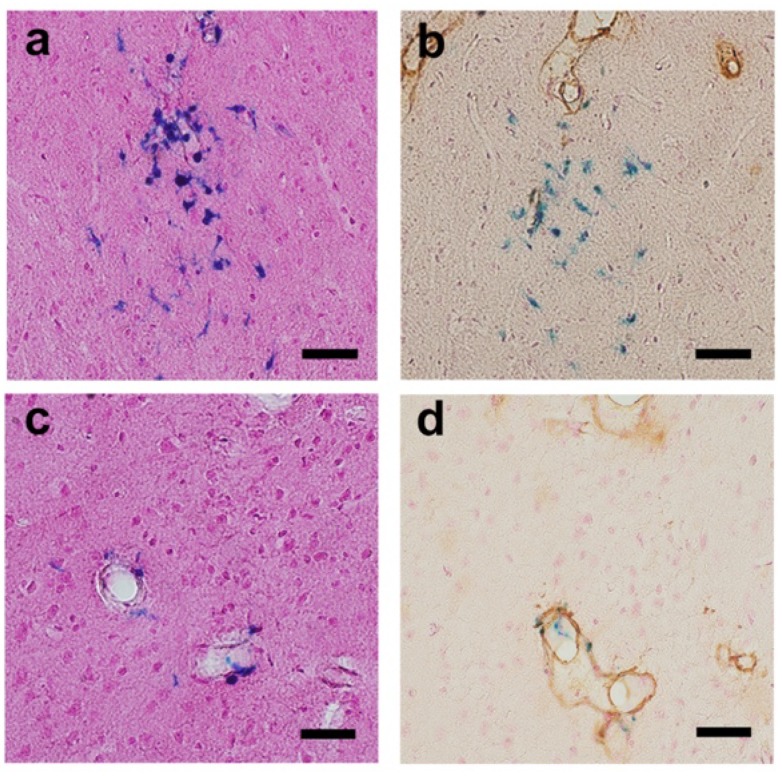
Representative images of cerebral micro-hemorrhage findings (a, b, Perls’s Berlin blue stain with Nuclear Fast Red (Kernechtrot stain solution)); b, d, double-labeled for amyloid (brown) and hemosiderin (blue)). (**a**) Clusters of hemosiderin staining are shown in the brain parenchyma (cortex at 1.35 mm behind Bregma). (**b**) In an adjacent section to a, some of them are in contact with amyloid-β (Aβ)-positive vessels. (**c**) Localized hemosiderin shown around the vessel wall (cortex at 0.85 mm behind Bregma). (**d**) In an adjacent section to c, localized bleeding to amyloid-laden vessels is shown. Scale bars indicate 10 µm.

**Figure 4:**
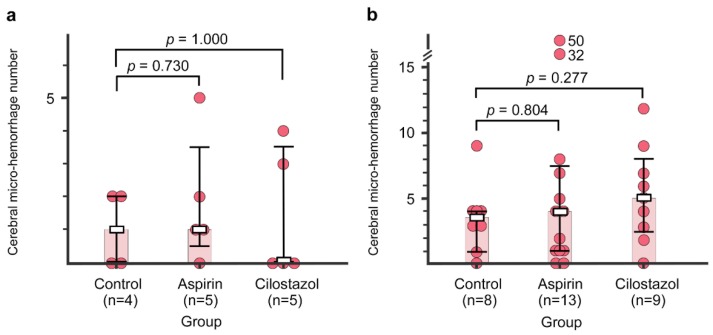
The number of cerebral micro-hemorrhages in the control, aspirin, and cilostazol groups evaluated at 15 months (**a**) and 21–23 months (**b**). Light-red-colored bar graphs indicate median. Boxes and bars indicate median and interquartile range, respectively. Red circles represent the number of cerebral micro-hemorrhages of each mouse. The numbers next to the red circles indicate the number of cerebral micro-hemorrhages, which are out of range of the vertical axis scale in two mice (b, Aspirin group).

The representative images of cerebral micro-hemorrhages are shown in Figure 3. In the first specimens (evaluated in mice aged 15 months), there were no differences in the number of cerebral micro-hemorrhages between the control group (median, interquartile range (IQR): 1, 0–2) and the aspirin group (1, 0.5–3.5) or the cilostazol group (0, 0–3.5) (*p* = 0.730 and *p* = 1.000, Mann–Whitney *U* test: Figure 4a). In the second specimens (evaluated in mice aged 21–23 months), there were also no differences in the number of cerebral micro-hemorrhages between the control group (3.5, 1.5–4) and the aspirin group (4, 1–7.5) or the cilostazol group (5, 2.5–8) (*p* = 0.804, and *p* = 0.277, Mann–Whitney *U* test, respectively: Figure 4b).

### 2.5. CAA Burden

**Figure 5:**
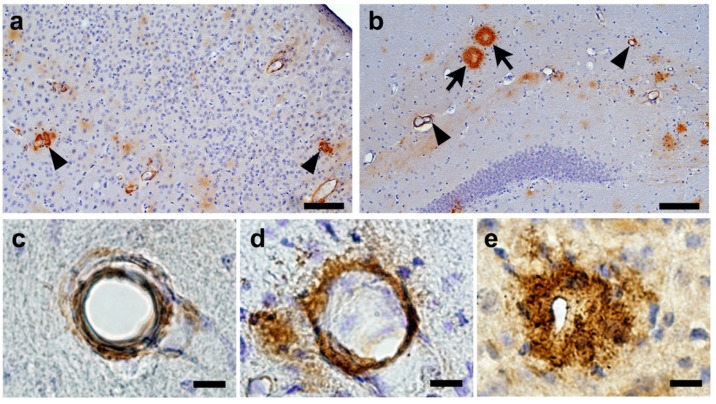
Representative images of cerebral amyloid angiopathy (CAA) in a Tg2576 mouse (aged 23 months old). Pan-Aβ immunostained sections show significant CAA in the cortex at 1.08 mm behind Bregma (a: arrowheads) and mild to moderate CAA (b: arrowheads) in the hippocampus at 1.33 mm behind Bregma. Arrows show senile plaques in the hippocampus (**b**). Vessel with a thin rim of amyloid in the vessel wall (c; severity grade, 1); vascular amyloid with amyloid infiltrating the surrounding neuropil (d; severity grade, 2); dysphoric amyloid with amyloid deposition within the vessel wall and with a thick and complete amyloid coat around the vessel wall (e; severity grade, 3). Scale bars indicate 100 µm (**a**,**b**) and 10 µm (**c**–**e**).

**Figure 6:**
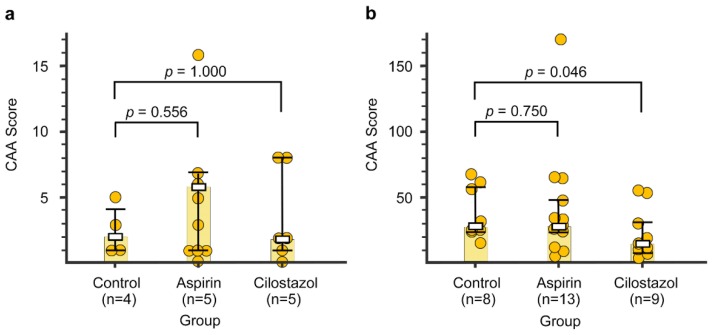
The cerebral amyloid angiopathy (CAA) scores in the control, aspirin, and cilostazol groups evaluated at 15 months (**a**) and 21–23 months (**b**). Light-yellow-colored bar graphs indicate median. Boxes and bars indicate median and interquartile range, respectively. Yellow circles represent the CAA score of each mouse.

The representative images of CAA burden are shown in Figure 5. In the first specimens, there were no differences in the CAA score between the control group (median, IQR: 2, 1–4) and the aspirin group (6, 1–7) or the cilostazol group (2, 1–8) (*p* = 0.556 and *p* = 1.000, Mann–Whitney *U* test: Figure 6a). In the second specimens, there was no difference (*p* = 0.750, Mann–Whitney *U* test) in the CAA score between the control group (28.5, 24.5–58) and the aspirin group (28, 23–47). Even after an outlier of the CAA score in the aspirin group (168) was excluded, non-significant results still remained (median, IQR: the aspirin group, 27, 18.5–40.5, *p* = 0.521, Mann–Whitney *U* test). In contrast, the CAA score of the cilostazol group (14, 11–31) was significantly lower in the control group (*p* = 0.046, Mann–Whitney *U* test: Figure 6b).

### 2.6. Senile Plaque

**Figure 7:**
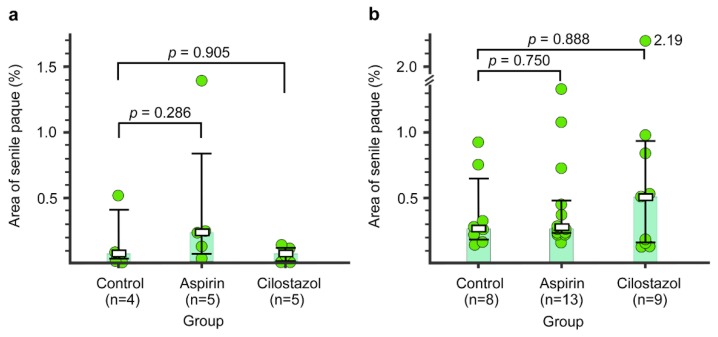
The percent area of senile plaque in the control, aspirin, and cilostazol groups evaluated at 15 months (**a**) and 21–23 months (**b**). Light-green-colored bar graphs indicate median. Boxes and bars indicate median and interquartile range, respectively. Green circles represent the value of percent area of senile plaque of each mouse. The numbers next to the green circle (2.19) indicate percent area of senile plaque out of the range of the vertical axis scale (b, Cilostazol group).

In the first specimens, there were no differences in the percent area of senile plaque between the control group (median, IQR: 0.05, 0.03–0.40) and the aspirin group (0.24, 0.08–0.83) or the cilostazol group (0.73, 0.00–0.12) (*p* = 0.286 and *p* = 0.905, Mann–Whitney *U* test: Figure 7a). In the second specimens, there were also no differences in the percent area of senile plaque between the control group (median, IQR: 0.27, 0.19–0.54) and the aspirin group (0.28, 0.22–0.45) or the cilostazol group (0.50, 0.16–0.84) (*p* = 0.750 and *p* = 0.888, Mann–Whitney *U* test: Figure 7b).

## 3. Discussion

The main finding of this study is that the CAA burden in Tg2576 mice could be reduced by half with long-term cilostazol therapy, but not with aspirin. In particular, such a long-term treatment effect on the CAA burden of APP transgenic mice was firstly archived with a lower dose of cilostazol (conducted to intake 20 mg/kg/day; resulting intake 14.7 mg/kg/day) compared to a previous study (600 mg/kg/day) [12]. However, we found no long-term treatment effect of either cilostazol or aspirin on the severity of cerebral micro-hemorrhages or on the expansion of senile plaque.

PDE family proteins, mostly expressed in the brain, have attracted attention as a source of new targets for the treatment of psychiatric and neurodegenerative disorders [13,14]. Previous studies of animal models have shown that the phosphodiesterase Ⅲ inhibitor, cilostazol, decreases cerebral amyloid-β accumulation [12,15]. Regarding the long-term effects of cilostazol on CAA, one study using Tg-SwDI mice mainly expressing vasculotropic Aβ demonstrated that relatively high-dose cilostazol (equivalent to 600 mg/kg/day intake in 25 g weight mouse) decreased the Aβ accumulation of the brain, resulting in improved cognitive performance [12]. Regarding the short-term effects of cilostazol, one study using C57BL/6J mice reported that the oral administration of cilostazol (20 mg/kg/day) for 6 weeks around the injection of Aβ into the cerebral ventricle almost completely prevented Aβ accumulation in the brain [15]. Thus, our finding has added one line of evidence that the efficacy of lower-dose cilostazol was replicated in a different experimental setting. Given the dose-dependent side effects (e.g., headache and dizziness) of cilostazol [16], long-term administration of the lower dose of cilostazol could be an optional strategy of the treatment for CAA burden.

The protective role of cilostazol against Aβ burden in the CAA model mice (i.e., Tg-SwDI mice or Tg2576 mice) appeared to be its vasculotropic effects, as long-term aspirin treatment did not reverse Aβ deposition. Our result of the reduced CAA score and non-reduced senile plaque suggests that the promotion of Aβ metabolism by vasculotropic cilostazol was achieved by its easy access to the perivascular area but not to the brain parenchyma. This is consistent with the finding that cilostazol poorly penetrates the blood–brain barrier [12]. The main mechanism promoting Aβ metabolism by cilostazol appears to be the increase in perivascular drainage of Aβ, followed by the decrease in degenerative changes in vascular walls with Aβ deposits [12]. Since the motive force for perivascular Aβdrainage appears to be generated by arterial pulsations [17,18], the direct action of cilostazol on the vascular smooth muscle cells to increase pulse duration time [19] and arterial elasticity [16] may have contributed to facilitating the perivascular drainage of Aβ.

None of the Aβ-targeted phase 3 clinical trials in Alzheimer’s disease has shown statistically significant benefits on its pre-specified clinical endpoints. Several of these trials, however, were mis-designed in terms of patient selection, choice of agent, target engagement, and/or dose, or they had to be halted because of the off-target side effects [20]. A recent phase 2 clinical trial in patients with CAA has shown that immunotherapy using the anti-Aβ40 antibody (Ponezumab) also did not show the prespecified efficacy (improvement in cerebrovascular reactivity measured by functional magnetic resonance imaging (MRI)) [21]. The Aβ-targeted immunotherapy could cause amyloid-related imaging abnormalities (ARIA) representing vasogenic edema, micro-hemorrhages, or cortical, superficial siderosis on MRI. ARIA, which appeared to be a dose-dependent phenomenon, sometimes causes transient symptoms of headaches, confusion, and visual disturbances [22]. Thus, it might not be realistic to increase the dose of the anti-Aβ antibody to enhance the effectiveness. To explore treatment to minimize Aβ accumulation, it might to be crucial to consider not only the suppression of Aβ over-production, but also the promotion of Aβ clearance in CAA and/or Alzheimer’s disease patients. A Japanese retrospective study reported that the combination therapy group (using donepezil plus cilostazol) was more effective for cognitive decline in patients with mild dementia compared to the donepezil-only group [23]. Thus, given that the balance between Aβ synthesis and clearance determines brain Aβ accumulation, a multidrug combination (e.g., low-dose anti-Aβ antibody, and low-dose cilostazol) therapy could provide a mainstream cure in the early stages of CAA and/or Alzheimer’s disease [24].

Our result that aspirin had no influence on the severity of cerebral micro-hemorrhages was in line with a previous study [25]. A characteristic feature of cilostazol is that it has weaker hemorrhagic side effects than other antiplatelet drugs [26] and does not increase the bleeding time [27]. In fact, a previous study demonstrated that the short-term administration of cilostazol (30 mg/kg/day) reduced the intracranial hemorrhage volume along with sufficient inhibition of platelet aggregation in non-transgenic mice [11]. However, the present study did not suggest the expected safety benefits of cilostazol for reducing micro-hemorrhages over aspirin. In other words, this study offered evidence that low-dose cilostazol could improve the vascular amyloid burden without increasing cerebral micro-hemorrhages in a mouse model of CAA.

We also acknowledge a limitation of this study. We did not evaluate the effects of lower-dose cilostazol on cognitive performance in Tg2576 mice. Although we had discussed this issue at the planning state of the study, we decided to just focus on whether lower-dose cilostazol could reduce the pathological burden of the brain in Tg2576 mice, because of our funding limitations to develop a research environment to fully examine the cognitive function of the mice (i.e., to buy additional Tg2576 mice or appropriate experimental devices). However, our current results allow us to believe that further investigation would be meaningful to calcify whether lower-dose cilostazol could improve cognitive performance in Tg2576 mice.

## 4. Materials and Methods

### 4.1. Standard Protocol Approval

All animal procedures were performed according to the guidelines of the Animal Use and Care Committee of the Saga University (Saga, Japan). All protocols were approved by the Animal Use and Care Committee and the Genetic Recombination Experimental Committee (ethical approval code: 23-024-2), as well as Animal Research: Reporting of In Vivo Experiments guidelines [28]. The experimental data is available from the supplemental materials: Data S1 and Data S2).

### 4.2. Animals

As a CAA animal model, we used female B6, SJL-Tg 2576Kha (APPSWE) transgenic mice (Tg2576 mice: Taonic Bioscience, Inc., NY, USA). The Tg2576 mice were housed in a room with a 12-h light/dark cycle (light on at 7:00 a.m.) with access to food and water ad libitum. A flow diagram of the study schedule and grouping is shown in Figure 1. Sixty Tg2576 mice aged 3 months were started on standard pelleted chow (vehicle). Eleven months after, mice aged 14 months were divided into three groups with adjustment for body weight: control group (mice fed with standard pelleted chow only), aspirin group (mice fed with the pelleted chow containing 0.01% aspirin), and cilostazol group (mice were fed with pelleted chow containing 0.01% cilostazol). The dose setting for each drug is described in the next section (please see 4.3, Drugs). The Tg2576 mice were randomly assigned to each group (in considering drug-associated death, we a priori planned to allocate more mice to the aspirin or the cilostazol group).

### 4.3. Drugs

In the brain of Tg2576 mice, Aβ deposits developed after 8 months, and dissemination of Aβ plaque progressed from 15 months to 23 months [29]. Therefore, we planned to evaluate the long-term effects of the drugs using mice aged 15 months or 21–23 months. For long-term administration of drugs, we selected oral drug intake using a pelleted chow containing each drug. The dose of cilostazol to be administered in mice varied because of the different bioavailability between humans and mice. A previous study reported that, in transgenic C57BL/6-Tg(Thy1-APPSwDutIowa) BWevn/J mice fed with pelleted chow containing 0.3% cilostazol (equivalent to 600 mg/kg/day intake in 25 g weight mouse), phosphodiesterase III inhibitor promoted the drainage of cerebrovascular Aβ [12], while a previous study demonstrated that in Aβ-injected wild-type mice, cilostazol administration of 10–20 mg/kg/day for 2 weeks exerted a protective effect against Aβ-induced cognitive deficits along with decreased Aβ accumulation [15]. To evaluate the effect of a lower dose of cilostazol on the CAA mice model, we used pelleted chow containing 0.01% cilostazol (equivalent to 20 mg/kg/day intake in 25 g weight mouse, donated by Otsuka Pharmaceutical, Tokyo, Japan) for treatment of the cilostazol group. Regarding the dose of aspirin, a previous study reported that, in spontaneous hypertensive rats, aspirin-attenuated collagen-induced platelet aggregation at 10–100 mg/kg in rats [30]. Clinically, daily intake of low-dose aspirin in humans is almost similar to cilostazol. Therefore, similar to the drug concentration of cilostazol, we used the pelleted chow containing 0.01% aspirin (equivalent to 20 mg/kg/day intake in 25 g weight mouse: donated by Otsuka Pharmaceutical, Tokyo, Japan) for treatment of the aspirin group.

### 4.4. Measurements of Estimated Individual Food Consumption and Drug Intake by the Groups

Food consumption per cage was measured once a week. Daily estimated individual food consumption (g/day) was calculated by the following formula: food consumption during a week per cage (g) / 7 (days) / the number of surviving mice in the cage. Daily estimated individual drug intake per weight of a mouse (mg/kg/day), which was also estimated per cage per week, was calculated by the following formula: the daily individual food consumption of the cage (g/day × 1000) × drug concentration/mean weight of a surviving mouse in the cage (kg).

### 4.5. Histology and Immunohistochemistry

To evaluate the serial effects of aspirin or cilostazol on Aβ deposition, pathological examinations were performed 1 month after the grouping (the first specimen, 15 months old, (*n* = 14)) and at 7–9 months after the grouping (the second specimen, 21–23 months old, (*n* = 30)). Mice were overdosed with sodium pentobarbital (50 mg/kg, intraperitoneal) and perfused transcardially with phosphate-buffered saline (PBS), followed by 4% paraformaldehyde in 0.1 M PBS. The brains were immediately removed, immersion fixed for 1 d in 4% paraformaldehyde, followed by 2 d in 10% sucrose in 0.01 M PBS, and 2 d in 30% sucrose in 0.01 M PBS. Post-fixed brains were cryoprotected, frozen, and sectioned at 25 μm with a freezing–sliding microtome [31]. Hematoxylin and eosin (H&E) and Congo red staining were done according to standard protocols [32]. Perls’s Berlin Blue method was used to visualize ferric iron in hemosiderin (with Nuclear Fast Red (Kernechtrot stain solution: Lot number, 130312: Muto Pure Chemicals Co., LTD., Tokyo, Japan)) [32,33]. According to previously published protocols [31,34], the pan-Aβ stain was performed with the following primary and secondary antibodies: the primary antibody, rabbit polyclonal antibody to Aβ (1–40) (catalog number, 44–136: Invitrogen by Thermo Fisher Scientific Inc., CA, USA); the secondary antibody, biotinylated goat anti-rabbit IgG antibody (catalog number, BA-1000: Vector Laboratories, CA, USA). For confocal microscopy, double-labeling for Aβ and smooth muscle cells was achieved simultaneously using frozen sections as follows: for Aβ, the primary antibody with rabbit polyclonal antibody to Aβ (1–40) (catalog number, 44–136: Invitrogen by Thermo Fisher Scientific Inc., CA, USA); the secondary antibody with Donkey anti-Rabbit IgG antibody Cy3 conjugate (1:500; product number, AP182C: Millipore Co., CA, USA); for smooth muscle cells, monoclonal anti-alpha-smooth muscle actin conjugate FITC (product number, F3777: Sigma-Aldrich Co. LLC, MO, USA). Sections were mounted with Fluoromount (catalog number: K 024: Diagnostic BioSystems, Hague, Netherlands) and analyzed with a Confocal Laser Scanning Microscope LSM880+Airyscan Fast (Zeiss, Oberkochen, Germany).

### 4.6. Pathological Evaluations

#### 4.6.1. Observation of Natural Changes of Cerebrovascular Amyloid Burden and Smooth Muscle Cell Loss

To confirm the findings of the age-related progression of cerebrovascular amyloid burden and accompanying smooth muscle cell loss in Tg2576 mice, mice fed with standard pelleted chow (i.e., control group) were evaluated at different times (15 months old and 23 months old) using confocal microscopy with double-labeling for Aβ and smooth muscle cell actin. We also evaluated with negative controls without primary antibody for Aβ (follow the same staining protocol without the addition of a primary antibody) to dismiss a possible age-dependent nonspecific stain for the secondary antibody.

#### 4.6.2. Specimens and Raters

All findings were evaluated through the cortex and the hippocampus of the right hemisphere. The ratings of hemorrhagic findings of the brain and any Aβ-positive vessels were evaluated by two raters (K.K. and K.U.), who were blinded to our hypothesis and information of the food content on each mouse. If results were different between the raters, the final decision was made after discussions by the two raters. CAA severity, described below, was classified by a single rater (K.K.) after the determinations of Aβ-positive vessels. Regarding senile plaque, quantitative analysis was performed with a semiautomatic computer-assisted processing system, as mentioned below, by a single rater (K.K.).

#### 4.6.3. Quantitation of Cerebral Hemorrhage(s)

Any acute subdural or cerebral bleeding(s) was defined as a large accumulation of erythrocytes in the intracranial space observed on the H&E stains with sets of systematically sampled sections (every 10th section throughout the cortex and the hippocampus (right hemisphere only)). Cerebral micro-hemorrhages, defined as clusters of hemosiderin staining on Perls’s Berlin blue stain with a delayed appearance of hemosiderin-positive microglia [35], located in the brain parenchyma and the around the vessel walls (Figure 3), were quantified on additional sets of every 10th section (right hemisphere only). The ratings of these findings were evaluated by two raters (K.K. and K.U.), who were blinded to our hypothesis and information of the food content on each mouse.

#### 4.6.4. Quantitative Analysis of CAA Burden

All quantification of CAA burden was done as previously published [31]. The frequency and severity of CAA were quantified on systematically sampled serial pan-Aβ immunostained sections throughout the region of interest (every 10th section through the cortex, and the hippocampus). Severity of CAA was classified by a single rater (K.K.), who were blinded to our hypothesis and the information of the food content on each mouse. “CAA frequency” was calculated by counting the total number of any Aβ-positive vessels in the entire set of systematically sampled sections. Regarding the CAA severity, all Aβ-positive vessels were classified into three grades (Figure 5) with a rating scale as described previously [36,37]: severity grade 1 = vessels with a thin rim of amyloid in the vessel wall; severity grade 2 = vascular amyloid with amyloid infiltrating the surrounding neuropil; severity grade 3 = dysphoric amyloid with amyloid deposition within the vessel wall and with a thick and complete amyloid coat around the vessel wall. The mean for all Aβ-positive vessels was taken as CAA severity. To evaluate comprehensive CAA burden, a “CAA score” was calculated by multiplying CAA frequency with CAA severity [31].

#### 4.6.5. Quantitative Analysis of Senile Plaque

**Scheme A1:**
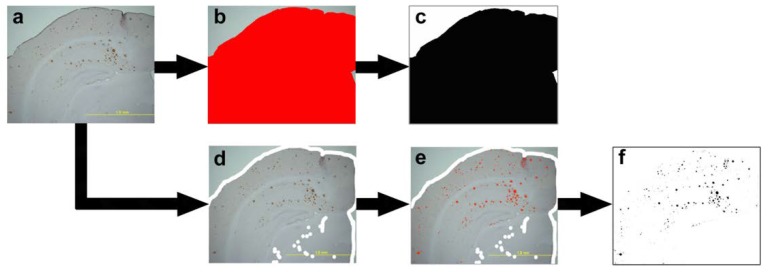
Semi-automatic measurement of senile plaques in the regions of interest (cortex and the hippocampus). Upper line: Measurement of the area of the section: (1) Filling of the section (**a**) with red color (**b**); (2) dichotomization of color into black and white using a semiautomatic method with an appropriate color threshold (**c**). Lower line: Measurement of the total area of the senile plaque: (1) digital stripping of Aβ-stained lesions located out of the regions of interest (**d**); (2) change of color of the remaining Aβ-stained area to red using manual calibration with an appropriate color threshold (**e**); (3) dichotomization of color to black (Aβ-stained lesions in regions of interest) and white (other) using a semiautomatic method with an appropriate color threshold (**f**).

Using a computer-assisted processing system (Image J version 1.49 for Mac; National Institutes of Health, Bethesda, MD, USA), the area of pan-Aβ stained lesions in the cortex and the hippocampus, corresponding to senile plaques, was quantified semi-automatically [38] (Scheme A1: please see appendix) by a single rater (K.K.), who was blinded to our hypothesis and the information of the food content on each mouse. Every section of the right hemisphere with Aβ stained electrically was converted to Joint Photographic Experts Group (JPEG) images with the same scale. These JPEG images were analyzed with Image J (version 1.49 for Mac: National Institutes of Health, Bethesda, MD, USA) with appropriate calibrations, as follows. Measurement of area of the section: (1) fill section with red color; (2) dichotomization of color for black and white using a semiautomatic method with an appropriate color threshold; (3) measurement of the black area. Measurement of total area of senile plaque: (1) digital stripping of Aβ-stained lesions located out of the regions of interest; (2) change color of the remaining Aβ-stained area to red using manual calibration with appropriate color threshold; (3) dichotomization of color for black (Aβ-stained lesions in regions of interest (i.e., cortex or hippocampus)) and white (other) using a semiautomatic method with an appropriate color threshold; (4) measurement of the black area. Thus, the total areas of the section, as well as total areas of Aβ-stained lesions, in the right hemisphere were quantitatively measured to count the pixels with a given intensity. To evaluate the degree of the senile plaque in brain parenchyma (including cortex and hippocampus), the percentage of senile plaque area in the brain (% area of senile plaque) was calculated with the following formula:

% senile plaque = total area of senile plaque / total area of the sections(1)

#### 4.6.6. Statistical Analyse

All statistical analyses were performed with two group comparisons (control group vs. aspirin group or cilostazol group) using the IBM SPSS statistics software program, version 21.0 (IBM, Armonk, NY, USA). As our variables (including cerebral micro-hemorrhages number, CAA score, and percent area of senile plaque) did not follow a normal distribution, a non-parametric test (Mann–Whitney *U* test) was used for the two group comparisons. Log-rank test was used to compare survival rates. *p* values < 0.05 were considered statistically significant.

## 5. Conclusions

The present study shows pathological evidence that CAA burden is reduced by cilostazol, even at a low dose. Cilostazol may provide a novel, promising therapeutic target for patients with CAA and/or Alzheimer’s disease, potentially in combination with early Aβ immunization therapy.

## Figures and Tables

**Table 1:** Estimated individual feed consumption and drug intake (per mouse) of the three groups.

	Control Group	Aspirin Group	Cilostazol Group
Measurements, times	75	77	75
Estimated individual food consumption, g/day (SD)	3.57 (0.40)	3.67 (0.47) ^a^	3.67 (0.44) ^b^
Estimated individual drug intake, mg/kg/day (SD)	NA	14.7 (1.9)	14.7 (1.8)

^a^*p* = 0.289 vs. control; ^b^ *p* = 0.324 vs. control (Mann–Whitney *U* test); NA = not applicable; SD = standard deviation.

## Supplementary Materials

Supplementary materials can be found at https://www.mdpi.com/1422-0067/21/7/2295/s1.

## Abbreviations

Aβ	amyloid-β
APP	amyloid-β Protein Precursor
CAA	cerebral amyloid angiopathy
H&E	hematoxylin and eosin
IQR	interquartile range
JPEG	joint photographic experts group
PBS	phosphate-buffered saline
PDE	phosphodiesterase
